# Evaluation of a protocol for remote identification of mosquito vector species reveals BG-Sentinel trap as an efficient tool for *Anopheles gambiae* outdoor collection in Burkina Faso

**DOI:** 10.1186/s12936-015-0674-7

**Published:** 2015-04-15

**Authors:** Marco Pombi, Wamdaogo M Guelbeogo, Maria Calzetta, N’Fale Sagnon, Vincenzo Petrarca, Vincenzo La Gioia, Alessandra della Torre

**Affiliations:** Dipartimento di Sanità Pubblica e Malattie Infettive, Università di Roma “Sapienza”, Rome, Italy; Centre National de Recherche et Formation sur le Paludisme, Ouagadougou, Burkina Faso; Dipartimento di Biologia e Biotecnologie, Università di Roma “Sapienza”, Rome, Italy; Ministero della Difesa - Stato Maggiore della Difesa, Rome, Italy

## Abstract

**Background:**

Feasibility and costs of monitoring efforts aimed to monitor mosquito species are strictly dependent on the presence of skilled entomologists directly in the field. However, in several contexts this is not possible or easy to organize, thus limiting the possibility to obtain crucial information on presence/abundance of potential disease vectors and of new invasive species. Digital imaging approaches could be extremely useful in the frame of medical entomology to overcome this limit. This work describes a surveillance approach to collect and morphologically identify host-seeking malaria vectors based on remote transmission of digital images of specimens collected with *ad hoc* modified traps.

**Methods:**

CDC light trap (CDC) and the BG-Sentinel trap (BG), both baited with BG-lure and CO_2_, were modified in order to have collected mosquitoes immobilized on a bi-dimensional surface. The performance of the two traps in the field was comparatively tested by Latin-square experiments in two villages of Burkina Faso under low and high mosquito densities. The efficiency of identifications based the inspection of digital images *versus* microscopic identifications of collected specimens was compared.

**Results:**

A total of 1,519 mosquitoes belonging to 16 species were collected, of which 88.5% were microscopically identified as *Anopheles gambiae s.l.* (mainly *Anopheles coluzzii*, 85.7%). During dry season BG collected 15 times more females than CDC outdoors, whereas indoors the BG collected 0.4 times less than CDC. During rainy season the ratio BG/CDC was 6.4 and 0.7 outdoors and indoors, respectively. The efficiency of digital images *versus* microscopic identifications of collected specimens was 97.9%, 95.6% and 81.5% for Culicidae, Anophelinae and *An. gambiae s.l.*, respectively.

**Conclusions:**

Results strongly encourage the use of BG-trap for collecting host-seeking *An. gambiae* particularly in the outdoor environment, providing new perspectives to the challenge of collecting this fraction of the biting population, whose epidemiological relevance is increasing due to the success of large-scale implementation of indoor malaria vector control strategies. Moreover, results show that the transmission of digital images of specimens collected by the *ad hoc* modified host-seeking traps efficiently allows identification of malaria vectors, thus opening the perspective to easily carry out mosquito monitoring also in the absence of entomologists directly in the field.

**Electronic supplementary material:**

The online version of this article (doi:10.1186/s12936-015-0674-7) contains supplementary material, which is available to authorized users.

## Background

In recent years, the technological improvements of digital devices, data storage capacities and computer performances have radically changed the way researchers use images to collect and store information for scientific purposes. In the study of life history traits of biological organisms, such progresses have enabled the tracking of individual behaviour, fecundity or growth trajectory on a fine time scale and over long periods, leading to the development of automated tools for the analysis of digital pictures of laboratory microcosms [1]. Moreover, semi-automated quantification of bi-dimensional traits for digital plant phenotyping has also been successfully developed [2]. In the field of museum biological collections, digital imaging approaches have been pursued to obtain improved high-throughput workflows for digitizing and providing access to invertebrate collections, opening the field to cybertaxonomy [3-6].

However, digital approaches have been so far limited to laboratory settings and never applied to field studies in the frame of which they could be extremely useful particularly for medical and agricultural entomology surveys. In fact, the presence in the field of specialized entomologist(s) is, nowadays, a *sine qua non* condition for surveillance/monitoring activities aimed to quickly obtain information about the presence of insect pests or of new invasive species. This need significantly complicates the logistic of the monitoring schemes and increases their costs. These issues could be overcome by a system relying on remote transmission of digital images of collected insect samples to a reference laboratory for species identification.

The first requirement to develop such as “digital identification system” is the immobilization of collected insect specimens on a bi-dimensional surface, such as a sticky-film. Several kinds of sticky-traps have already been developed and employed in the field to passively collect mosquito vectors at different physiological stages. The collection of gravid mosquitoes with adhesive ovitraps has been exploited in several studies, most of which targeting *Aedes aegypti, Aedes albopictus*, *Culex pipiens*/*quinquefasciatus* and other container-breeding mosquitoes in tropical as well as in temperate areas [7-15]. The Sticky Resting Box has proved efficient for collecting resting *Anopheles gambiae*, both in indoor and outdoor environments in Burkina Faso [16]. Sticky-sheets floating over breeding sites have proved effective for collecting ovipositing *Anopheles* females and other Culicidae in Tanzania [17]. A wire mesh glue trap with fruits/seedpods suspended on skewers inside has been used to collect sugar-seeking *An. gambiae* Mali [18]. However, none of these sticky-traps targets host-seeking mosquito females, despite the fact that these represent the most epidemiologically important fraction of vector populations.

This work reports the development of an approach for the remote monitoring of host-seeking mosquito vectors that has been tested in a highly endemic malaria area of West Africa. This approach implied the *ad hoc* modification of two traps routinely used to collect host-seeking mosquito females - i.e. the CDC light trap and the BG-Sentinel trap – in order to directly collect mosquitoes immobilized on a bi-dimensional surface for subsequent digital morphological identification.

## Methods

### Trap descriptions

**Figure 1:**
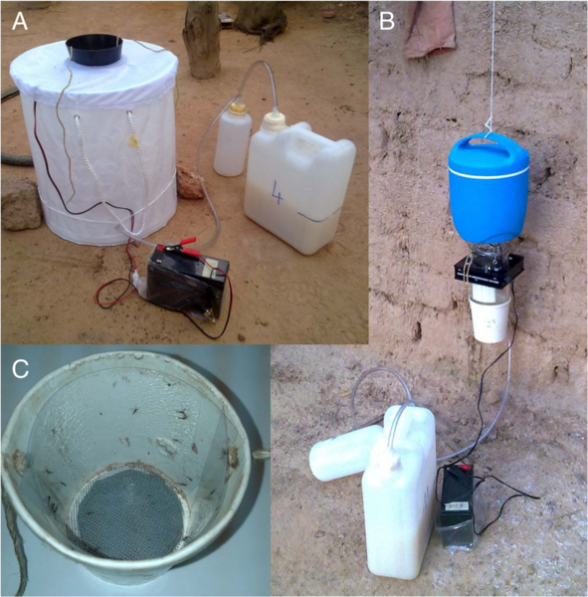
Field set-up of the two collection methods evaluated in the study. **A)** BG-Sentinel equipped with sticky-cup replacing the collection bag in its upper entrance and BG-lure + CO_2_ produced by fermenting yeasts; **B)** CDC light trap equipped with sticky-cup replacing the collection bag at the bottom and BG-lure + CO_2_ produced by fermenting yeasts; **C)** detail of a sticky-cup after mosquito collection showing the two inner sticky panels at its sides and the sticky net at its bottom.

BG-Sentinel (Biogents GmbH, Regensburg, Germany, hereafter BG) and CDC light (model IMT, PeP, San Giuliano Milanese, Italy, hereafter CDC) traps were modified in order to collect the mosquitoes entering the traps in sticky-cups replacing the standard bags (Figure 1A and B). The sticky-cup consists of a white cardboard paper soup cup (Ø 10 cm, Figure 1C) whose basis is replaced by a net in order not to block the air flow produced by the trap fan. The net and two transparent panels lining the inner walls of the cup are coated with rat glue (Zapicol; Zapi Industrie Chimiche SpA, Conselve, Padova, Italy). The glue is formulated in order to maintain its adhesive properties for a long time even under extreme heat and humidity conditions [16]. Both the BG and the CDC traps were baited with the commercial Biogents BG-lure and with a source of CO_2_ produced by sugar-fermenting yeast [19].

### Experimental design

A total of five 4x4 Latin-square experiments were carried out in the villages of Goden and Koubri (Ouagadougou area, Burkina Faso) during rainy (13–16 September 2011, 10–13 and 17–20 September 2012) and dry (4–10 and 16–19 April 2012) seasons in four family compounds/village. In each compound, four traps (two BG and two CDC traps) were rotated daily from two indoor and two outdoor positions, inside and outside two inhabited houses, respectively, for a total of 16 rotations/Latin square and 320 trap/days. Traps were activated for 12 hours starting at 6:00 PM. Batteries and CO_2_ source were replaced daily.

### Mosquito identification and evaluation of digital identification protocol

**Figure 2:**
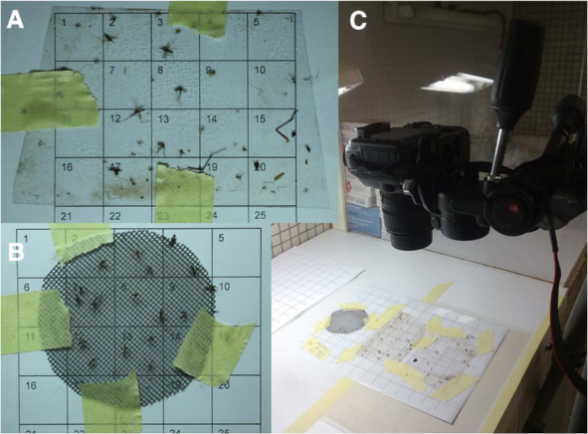
Details of the protocol for digital image acquisition of specimens collected by sticky surfaces. **A)** sample of a lateral sticky panel prepared for image acquisition; **B)** sample of a sticky net prepared for image acquisition; **C)** example of an image acquiring session using a Canon 5D Mark II + Canon EF 100 mm f/2.8 macro USM system.

Sticky-cups were brought to the laboratory and stuck mosquitoes were morphologically identified, separated by species, gender and gonotrophic stage under a stereomicroscope [20]. In a single Latin-square experiment (13–16 September 2011), digital pictures of the two internal panels (14x8cm, Figure 2A) and of the basis (Ø 7 cm, Figure 2B) of the sticky-cup were taken by a Canon 5D Mark II + Canon EF 100 mm f/2.8 macro USM system (Figure 2C). The shooting configuration used was ISO-100 (to minimize the digital noise), f/11 (to maximize the field depth), and the live view on during shooting (to eliminate the micro-shakes due to the movement of the mirror). The use of a flash was not needed and the image was taken using a common table lamp. Metadata about shooting parameters are available in Exif information embedded in exemplificative pictures available as Additional files 1, 2, and 3.

The digital pictures were visually inspected in order to identify genus, species and gender of collected specimens. Digital identifications were confirmed by microscopic identifications. *Anopheles gambiae s.l.* specimens were isolated cutting the sticky-surfaces around them and dipping these in acetone for two minutes. Isolated mosquitoes were stored in tubes containing silica-gel for subsequent molecular species identification by PCR-SINE X protocol [21].

### Statistical analysis

Variation in mosquito abundance between collection methods was investigated using Generalized Linear Mixed Models [22]. In these analyses, the sampling method was fitted as a main effect, with season, village, compound, house and date fitted as random effects. Mosquito abundance data are typically overdispersed, consequently a Poisson or Negative Binomial distribution (with or without zero-inflation) were tested to model the data, choosing a Negative Binomial distribution in all cases (with fit being assessed by both Likelihood Ratio Test and Akaike Information Criterion). The predicted mean numbers of female mosquitoes of *An. gambiae* complex species (i.e. *Anopheles coluzzii*, *An. gambiae s.s.*, *Anopheles arabiensis*) collected by each trapping method were obtained by extracting and exponentiating the coefficients and associated standard errors predicted from statistical models. All statistical analyses were conducted in R, v3.1.1 [23] using the lme4 [24] and glmmADMB [25] packages.

## Results and discussion

This work presents an approach to collect and remotely identify host-seeking mosquitoes in field settings where the need of expert entomologists carrying out morphological identification may raise feasibility and budget concerns. To this aim, two types of traps widely used to collect host-seeking mosquitoes (i.e. the BG-Sentinel and CDC light trap) have been equipped within a sticky-cup fit for immobilizing collected specimens on a bi-dimensional surface suitable for digital image acquisition. The performance of the two traps in two villages of Burkina Faso was compared both in indoor and outdoor environments, assessing the efficiency of the identifications based on digital images with that of microscopic identification, taken as reference method.

A total of 1,519 mosquitoes belonging to 16 species was collected and 1,345 (88.5%) of these were microscopically identified as *Anopheles gambiae s.l.* Other species collected were mostly *Culex decens* (3.8%), *Anopheles coustani* (2.8%), and *Cx. quinquefasciatus* (1.6%, see Additional file 4). Ninety-eight percent of *An. gambiae s.l.* specimens were successfully molecularly identified as *An. coluzzii* (85.7%), *An. arabiensis* (7.1%) and *An. gambiae s.s.* (6.8%). Three *An. coluzziiXgambiae* and one *An. coluzziiXarabiensis* hybrids were also identified.

**Figure 3:**
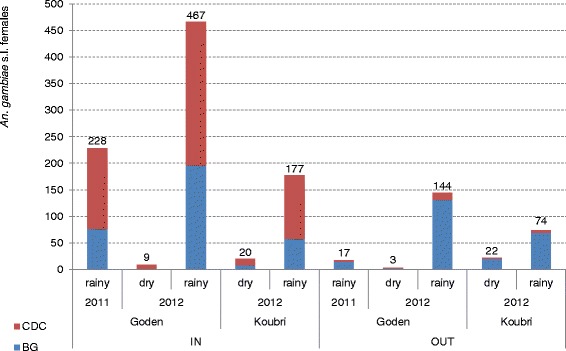
*Anopheles gambiae* s.l. females collected with BG and CDC traps indoors and outdoors. Total numbers of *Anopheles gambiae* s.l. females collected with BG-Sentinel (blue bars) and CDC light trap (red bars) indoors and outdoors separated per village, sampling yeas and season. BG = BG-Sentinel trap; CDC = CDC light trap; IN = indoor sampling; OUT = outdoor sampling.

Overall, collections of *An. gambiae s.l.* females were consistently higher in BG outdoors and in CDC indoors (Figure 3). This is clearly revealed by the results of the GLMM models for each of the three *An. gambiae* complex species captured (Table 1). In the case of the most abundant species, *An. coluzzii*, BG trap collected 8.4 times more females than CDC trap outdoors (estimated mean of females collected per trap/night: 1.94 and 0.23 for BG and CDC, respectively; P < 0.001), whereas the opposite was observed indoors, with a BG/CDC ratio of 0.5 (BG = 2.0, CDC = 3.64; P = 0.01). A similar trend was observed for the other two taxa of the *An. gambiae* complex, *An. gambiae s.s.* and *An. arabiensis*, although the low frequency of these species in the study area did not allow highlighting significant differences.

**Table 1:** **Mean estimates of** ***Anopheles gambiae*** **complex species collected by BG and CDC traps**

**Species**	**Position**	**GLMM estimates**	**BG**	**CDC**	**P-value**	**Ratio**
*An. coluzzii*	OUT	mean	1.94	0.23	<0.001***	8.4
		2.5% c.l.	1.00	0.10		
		97.5% c.l.	3.77	0.51		
	IN	mean	2.00	3.64	0.01*	0.5
		2.5% c.l.	0.90	1.66		
		97.5% c.l.	4.44	7.97		
*An. gambiae s.s.*	OUT	mean	0.11	≃0	0.98	>100
		2.5% c.l.	0.26	0		
		97.5% c.l.	0.48	+∞		
	IN	mean	0.71	1.23	0.29	0.6
		2.5% c.l.	0.22	0.38		
		97.5% c.l.	2.33	4.00		
*An. arabiensis*	OUT	mean	≃0	≃0	0.24	≃1
		2.5% c.l.	0	0		
		97.5% c.l.	+∞	+∞		
	IN	mean	0.19	0.27	0.34	0.7
		2.5% c.l.	0.07	0.09		
		97.5% c.l.	0.53	0.75		

Estimated means and confidence intervals of mosquito females/trap/night of *An. coluzzii*, *An. gambiae* s.s. and *An. arabiensis* collected by the two sampling methods baited with BG-lure + CO_2_ calculated by GLMM. BG = BG-Sentinel trap; CDC = CDC light trap; IN = indoor sampling; OUT = outdoor sampling; * significant difference; *** highly significant difference.

The separate analysis of *An. coluzzii* collected during dry (i.e. low-mosquito density) and rainy (i.e. high-density) seasons further highlights the differences observed in the overall analysis (Table 2). During dry season BG collected 15 times more females than CDC outdoors (estimated mean of females collected per trap/night: 0.15 and 0.01 for BG and CDC, respectively; P = 0.002), whereas indoors the BG collected 0.4 times less than CDC (BG = 0.24, CDC = 0.64; P = 0.008). During rainy season the differences were less sharp: BG collected 6.4 times more mosquitoes than CDC outdoors (BG = 3.19, CDC = 0.50; P < 0.001), and 0.7 times less indoors (BG = 7.23, CDC = 11.00; P = 0.08).

**Table 2:** **Mean estimates of** ***Anopheles coluzzii*** **collected by BG and CDC traps during rainy and dry seasons**

**Species**	**Season**	**Position**	**GLMM estimates**	**BG**	**CDC**	**P-value**	**Ratio**
*An. coluzzii*	dry	OUT	mean	0.15	0.01	0.002*	15
			2.5%	0.03	≃0		
			97.5%	0.68	0.11		
		IN	mean	0.24	0.64	0.008*	0.4
			2.5%	0.08	0.27		
			97.5%	0.68	1.51		
	rainy	OUT	mean	3.19	0.50	<0.001***	6.4
			2.5%	1.61	0.24		
			97.5%	6.30	1.05		
		IN	mean	7.23	11.00	0.08	0.7
			2.5%	4.32	6.56		
			97.5%	12.11	18.40		

Estimated means and confidence intervals of mosquito females/trap/night of *An. coluzzii* collected during rainy and dry seasons by the two sampling methods baited with BG-lure + CO_2_ calculated by GLMM. BG = BG-Sentinel trap; CDC = CDC light trap; IN = indoor sampling; OUT = outdoor sampling; * significant difference; *** highly significant difference.

These results are the first evidence from a field study showing that BG-Sentinel trap can be effectively exploited to collect host-seeking *An. gambiae* mosquitoes, at least when baited with BG-lure and CO_2_. In fact, outdoors BG trap performance was shown to be largely superior to equally baited CDC trap both in high and low mosquito density conditions, while indoors it remained effective although with lower performance compared to CDC trap. Notably, the numbers of *An. coluzzii* females collected per trap/night by BG trap were in the same ranges of those collected indoor by back-pack aspirations and outdoors by aspiration in pit-shelter aspirations in the same villages and seasons [16].

A total of 68 images with 287 mosquitoes were visually inspected and results were compared to those obtained by microscopic identification, resulting in overall success rates of 97.9% for Culicidae (281/287), 95.6% Anophelinae (262/274), 81.5% for *An. gambiae s.l.* (212/260) and in a success rate per single daily trap capture ranging from 75% to 100%. The images collected by capture system here described demonstrated to be detailed enough to obtain good identifications (see Additional files 1, 2, and 3). If needed, a more sensitive system could be applied, increasing the image sensor performance (i.e. more than 21Mpixels) and/or the magnification rate of lens (i.e. more than 1:1). However, the latter solution have the limit to risk to not cover the whole sticky surface, thus needing the acquisition of more than one image per sample. It is also possible to adopt the digital image identification protocol without using the glue to collect the specimens. In this case the sample collected with the classic bag can be placed on a white surface and them photographed, thus obtaining images of specimens not altered by the glue. However, the glue has the advantage to collect specimens that do not need to be killed in the field (by chloroform, ether or freezing) prior to be photographed. Moreover the image capture of glued specimens is not disturbed by the presence of wind which could move or disperse the specimens during the process. Also, the glue has the advantage to prevent possible power supply breakdowns that would accidentally allow captured insects to escape from the trap.

The type of trap used for collecting the specimens on the sticky surface (BG vs CDC) did not affect significantly species identification (Pearson’s Chi-square = 1.39, P = 0.24). This means that the position of the suction fan, which is placed after the sticky-cup in BG and before it in CDC, does not alter the quality of the collected sample. The high success rate of microscopic and digital identifications of specimens collected in sticky-cups may represent a general advantage of these modified trapping tools with respect to the unmodified traps in which the specimens are collected inside a bag. In fact, the continuous air circulation generated by the fan and the simultaneous collection of other bigger insects (e.g. moths) inside the bag may alter or even destroy diagnostic characters of mosquitoes collected, while specimens immobilized in the sticky-cup are protected from this inconvenient. The results also confirm that the glue used to immobilize the mosquitoes does not have a repellent effect, as already shown in other studies where the same glue was used on different sticky trap models to collect *An. gambiae*, *Cx. pipiens*, *Ae. albopictus* and *Ae. aegypti* in Africa, Europe and Asia [7,16,26-28].

## Conclusions

Results strongly encourage the use of BG-trap - which is quickly becoming the new gold standard for collecting host-seeking Dengue vectors - also for collecting host-seeking *An. gambiae* particularly in the outdoor environment. In fact, outdoor collection of host-seeking malaria vectors have been traditionally carried out by human landing catches which are nowadays considered unethical because of the risk of exposure of human volunteers to mosquito–borne diseases. On the other hand, CDC light-trap, which is the gold standard for indoor trapping, has shown weak efficacy outdoors [29] and little information is presently available on the outdoor performance of more recently developed traps, such as MM-X and SUNA traps, which have been comparatively tested against other traps only in semi-field conditions [30-32]. The shown efficacy of BG-trap outdoors provides new perspectives to the challenge of collecting the outdoor biting fraction of the malaria vectors populations, whose epidemiological relevance is increasing due to the success of large-scale implementation of indoor malaria vector control strategies [33].

Finally, the results here obtained demonstrate that the transmission of digital images of mosquito specimens efficiently allows “remote identification” of malaria vectors, thus opening the perspective to carry out mosquito monitoring also in the absence of entomologists directly in the field. This “remote” monitoring system could be in principle further expanded to target all fractions of a vector population, exploiting already available sticky-traps for ovipositing [7,8,10,11,17], resting [16] and sugar-feeding [18] mosquitoes, as well as modifying other traps with sticky surfaces, as done in the present study.

## Additional files

Additional file 1:
**Example of a picture used for mosquito morphological identification.** The image has been obtained photographing a sticky sheet of a CDC collection. It could be identified 9 females of *Anopheles gambiae* s.l. in the quarters 4, 5, 6, 8, 9, 10. Exif information containing shooting parameters are embedded in the file. Additional file 2:
**Example of a picture used for mosquito morphological identification.** The image has been obtained photographing a sticky sheet of a CDC collection. It could be identified one female of *Anopheles coustani* in the quarter 8. Exif information containing shooting parameters are embedded in the file. Additional file 3:
**Example of a picture used for mosquito morphological identification.** The image has been obtained photographing a sticky sheet of a CDC collection. It could be identified one female of *Culex nebulosus* in the quarter 3. Exif information containing shooting parameters are embedded in the file. Additional file 4:
**Total numbers of Culicidae specimens collected by BG and CDC traps separated per species and gender.** Numbers of female and male mosquitoes of different species collected by BG-Sentinel and CDC light trap during the different sampling years, seasons, villages and positions. BG= BG-Sentinel trap; CDC= CDC light trap; IN= indoor sampling; OUT= outdoor sampling; ♀= females; ♂= males.
