# Supplementary figures and images for: Evaluation of a protocol for remote identification of mosquito vector species reveals BG-Sentinel trap as an efficient tool for Anopheles gambiae outdoor collection in Burkina Faso
Source: Malar J. 2015 Apr 15;14:161. doi: 10.1186/s12936-015-0674-7 (PMC4406007; doi:10.1186/s12936-015-0674-7)

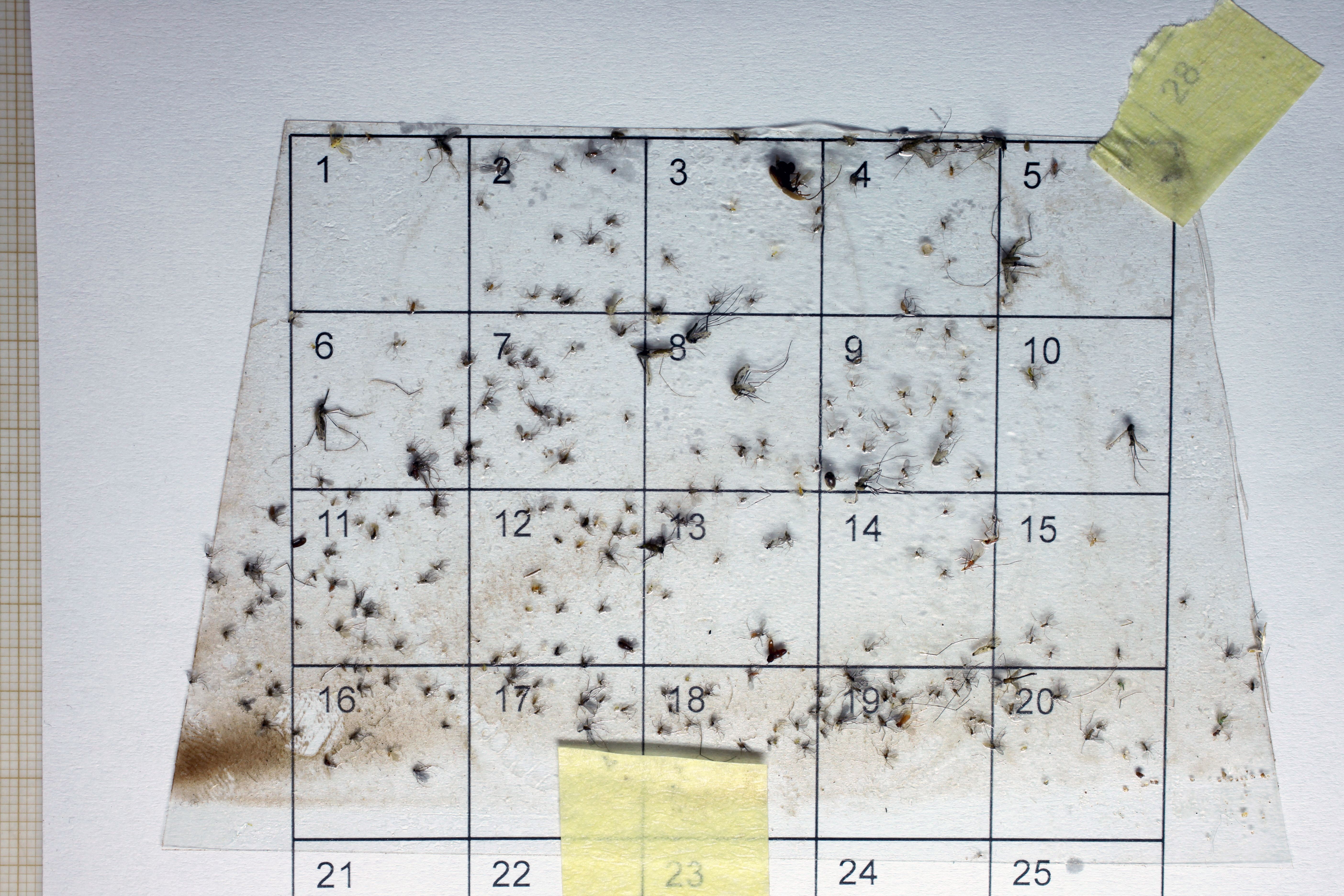

Supplement: Additional file 1: — Example of a picture used for mosquito morphological identification. The image has been obtained photographing a sticky sheet of a CDC collection. It could be identified 9 females of Anopheles gambiae s.l. in the quarters 4, 5, 6, 8, 9, 10. Exif information containing shooting parameters are embedded in the file. [file 12936_2015_674_MOESM1_ESM.jpeg]

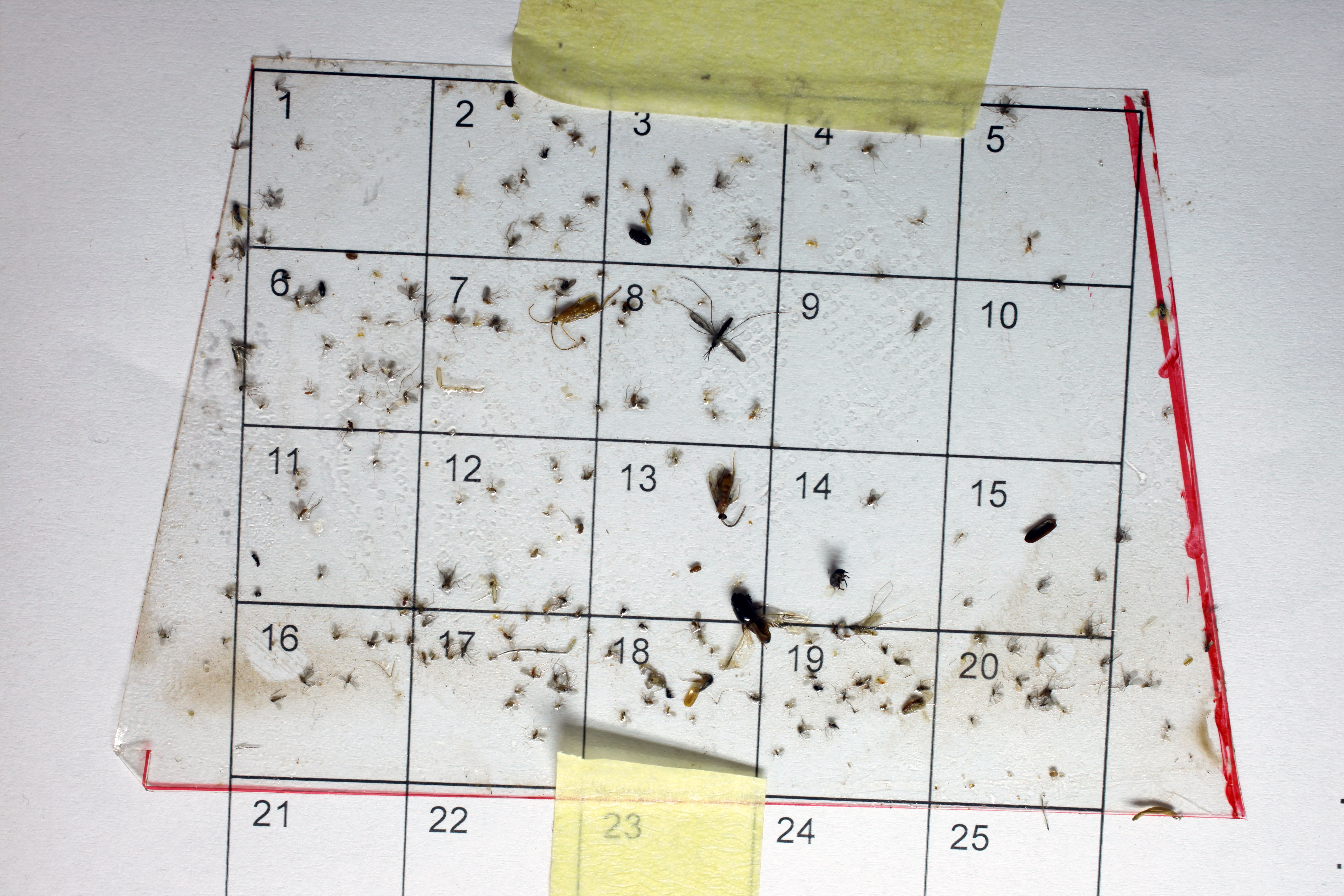

Supplement: Additional file 2: — Example of a picture used for mosquito morphological identification. The image has been obtained photographing a sticky sheet of a CDC collection. It could be identified one female of Anopheles coustani in the quarter 8. Exif information containing shooting parameters are embedded in the file. [file 12936_2015_674_MOESM2_ESM.jpeg]

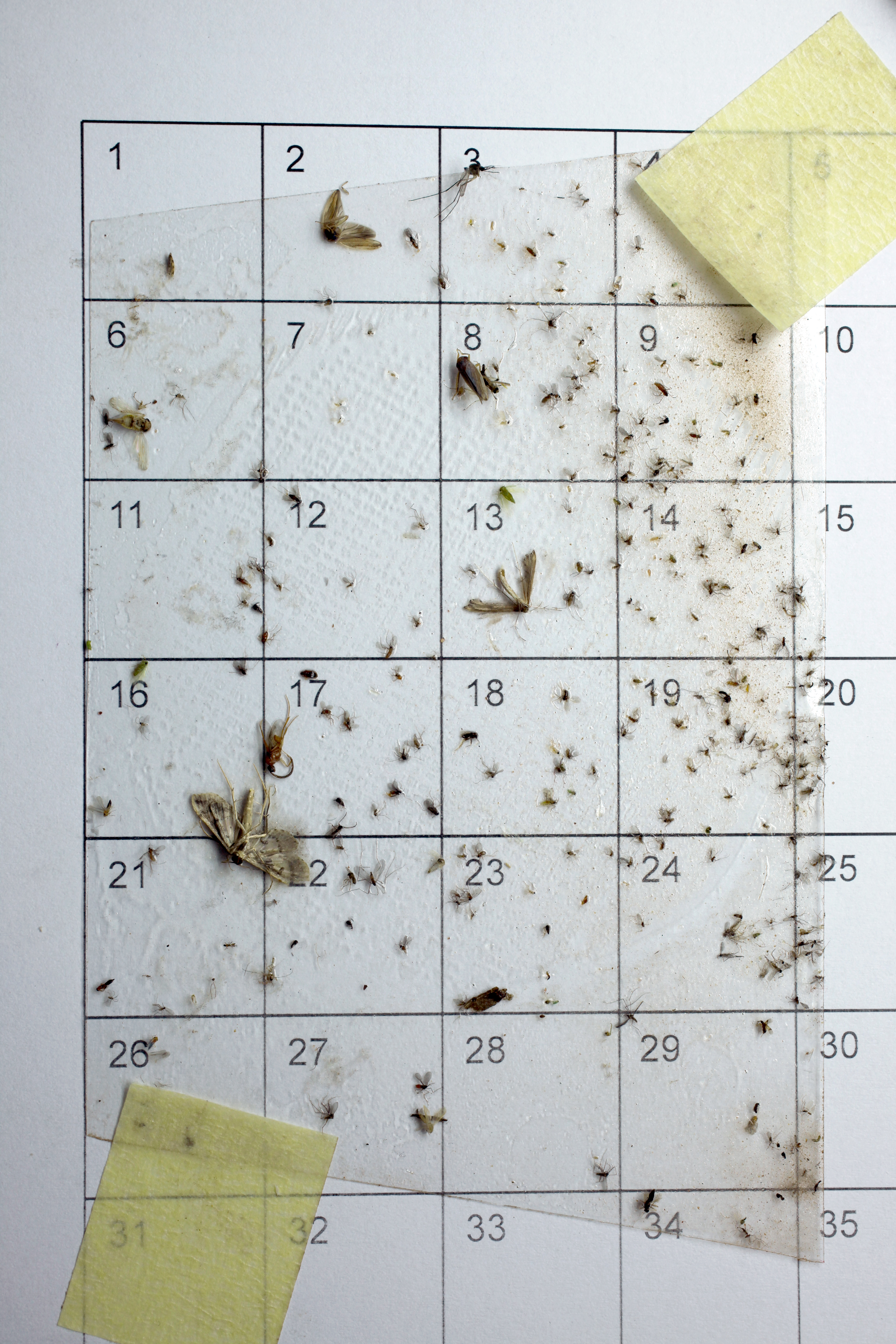

Supplement: Additional file 3: — Example of a picture used for mosquito morphological identification. The image has been obtained photographing a sticky sheet of a CDC collection. It could be identified one female of Culex nebulosus in the quarter 3. Exif information containing shooting parameters are embedded in the file. [file 12936_2015_674_MOESM3_ESM.jpeg]
