# Supplementary material for: Evaluation of a protocol for remote identification of mosquito vector species reveals BG-Sentinel trap as an efficient tool for Anopheles gambiae outdoor collection in Burkina Faso
Source: Malar J. 2015 Apr 15;14:161. doi: 10.1186/s12936-015-0674-7 (PMC4406007; doi:10.1186/s12936-015-0674-7)
Supplement: Additional file 4: — Total numbers of Culicidae specimens collected by BG and CDC traps separated per species and gender. Numbers of female and male mosquitoes of different species collected by BG-Sentinel and CDC light trap during the different sampling years, seasons, villages and positions. BG= BG-Sentinel trap; CDC= CDC light trap; IN= indoor sampling; OUT= outdoor sampling; ♀= females; ♂= males. [file 12936_2015_674_MOESM4_ESM.pdf]

Year	Season	Village	Position	Trap	An. gambiae s.l.		An. coustani		An. zeimanni		An. pharoensis		An. nili		An. funestus		An. rufipes		Cx. decens		Cx. quinquefasciatus		Cx. nebulosus		Cx. tigripes		Cx. cinereus		Ae. aegypti		Ae. taylori		Ae. hirsutus		Ma. uniformis		Culex sp.	Aedes sp.	Mansonia sp.
					♀	♂	♀	♂	♀	♂	♀	♂	♀	♂	♀	♂	♀	♂	♀	♂	♀	♂	♀	♂	♀	♂	♀	♂	♀	♂	♀	♂	♀	♂	♀	♂			
2011	rainy	Goden	IN	BG	77	2	1	0	0	0	0	0	0	0	1	0	0	0	0	0	0	0	0	0	0	0	0	0	0	0	0	0	0	0	0	0	0	0	0
				CDC	151	13	0	0	0	0	0	0	0	0	0	0	1	0	0	0	0	0	0	0	0	0	1	0	0	0	0	0	0	0	0	0	3	4	0
			OUT	BG	14	0	0	0	0	0	0	0	0	0	0	0	0	0	0	0	0	0	1	0	0	0	0	0	1	0	0	0	0	0	0	0	0	1	0
				CDC	3	0	3	0	4	1	2	0	1	0	0	0	0	0	0	0	0	0	1	0	0	0	0	0	0	0	1	0	0	0	0	0	0	0	0
2012	rainy	Goden	IN	BG	196	9	0	0	0	0	0	0	2	0	0	0	0	0	0	0	0	0	0	0	0	0	0	0	0	0	0	0	0	0	0	0	0	0	0
				CDC	271	1	0	0	0	0	0	0	1	0	0	0	0	0	0	0	0	0	0	0	0	0	0	0	0	0	0	0	0	0	0	0	0	0	0
			OUT	BG	131	12	0	0	0	0	0	0	1	0	0	0	0	0	0	0	0	0	4	0	0	0	0	0	0	0	0	0	0	0	0	0	0	0	0
				CDC	13	0	0	0	0	0	0	0	0	0	1	0	0	0	0	0	0	0	0	0	0	0	0	0	0	0	0	0	0	0	0	0	0	0	0
		Koubri	IN	BG	58	39	0	0	0	0	0	0	0	0	0	0	0	0	4	0	0	0	0	0	0	0	0	0	0	0	0	0	0	0	0	0	1	0	0
				CDC	119	34	5	0	0	0	2	1	0	0	0	0	0	0	0	0	0	0	0	0	0	0	0	0	0	0	0	0	0	0	0	0	0	0	4
			OUT	BG	69	39	2	0	0	0	1	0	0	0	0	0	0	0	5	0	4	1	0	0	1	0	0	0	1	0	0	0	1	0	0	0	0	0	0
				CDC	5	0	24	0	2	0	0	0	0	0	0	0	0	0	0	0	0	0	0	0	2	0	0	0	0	0	0	0	0	0	0	0	0	0	0
	dry	Goden	IN	BG	0	0	0	0	0	0	0	0	0	0	0	0	0	0	0	0	0	0	0	0	0	0	0	0	0	0	0	0	0	0	0	0	0	0	0
				CDC	9	6	0	0	0	0	0	0	0	0	0	0	0	0	2	1	0	0	0	0	0	0	0	0	0	0	0	0	0	0	0	0	0	0	0
			OUT	BG	2	0	0	0	0	0	0	0	0	0	0	0	0	0	1	0	0	0	0	0	0	0	0	0	0	0	0	0	0	0	0	0	0	0	0
				CDC	1	0	0	0	0	0	0	0	0	0	0	0	0	0	2	0	0	0	0	0	0	0	0	0	0	0	0	0	0	0	0	0	0	0	0
		Koubri	IN	BG	8	13	0	0	0	0	0	0	0	0	0	0	0	0	4	1	1	0	0	0	0	0	0	0	0	0	0	0	0	0	0	0	0	0	0
				CDC	12	8	4	1	0	0	0	0	0	0	0	0	0	0	12	4	5	0	0	0	0	0	0	0	0	0	0	0	0	0	2	0	0	0	0
			OUT	BG	20	1	1	0	0	0	0	0	0	0	0	0	0	0	10	1	9	2	0	0	0	0	0	0	0	0	0	0	0	0	0	0	0	0	0
				CDC	2	8	1	0	0	0	0	0	0	0	0	0	0	0	9	2	2	0	0	0	0	0	0	0	0	0	0	0	0	0	0	0	0	0	0
Total					1161	185	41	1	6	1	5	1	5	0	2	0	1	0	49	9	21	3	6	0	3	0	1	0	2	0	1	0	1	0	2	0	4	4	4
